# TBCancer: A database for exploring characteristics and functions of tissue‐biased genes in cancer

**DOI:** 10.1002/imo2.70039

**Published:** 2025-06-29

**Authors:** Zhuobin Lin, Kunhua Hu, Hongyan Sun, Xiaoqiong Bao, Lin Tang, Wei Liu, Zhixiang Zuo, Zhihang Chen

**Affiliations:** ^1^ Guangdong Key Laboratory of Liver Disease Research, The Third Affiliated Hospital of Sun Yat‑sen University Sun Yat‐sen University Guangzhou China; ^2^ State Key Laboratory of Oncology in South China, Cancer Center, Collaborative Innovation Center for Cancer Medicine Sun Yat‐sen University Guangzhou China; ^3^ School of Life Sciences Sun Yat‐sen University Guangzhou China; ^4^ Department of Interventional Medicine The Fifth Affiliated Hospital of Sun Yat‐sen University Zhuhai China

**Keywords:** database, immune escape, multi‐omics, pan‐cancer, stemness, tissue‐biased gene

## Abstract

This study defined 10,921 tissue‐biased genes across 54 normal tissues and 41 cancer types. Tumor‐associated tissue‐biased genes exhibit downregulation, mutations, and epigenetic modifications, correlating with poor clinical outcomes. Their inactivation promotes tumorigenesis by enhancing stemness and immune evasion, highlighting their value as prognostic biomarkers and therapeutic targets. To facilitate research, we developed a database integrating multi‐omics data on these genes for mechanistic and therapeutic exploration.

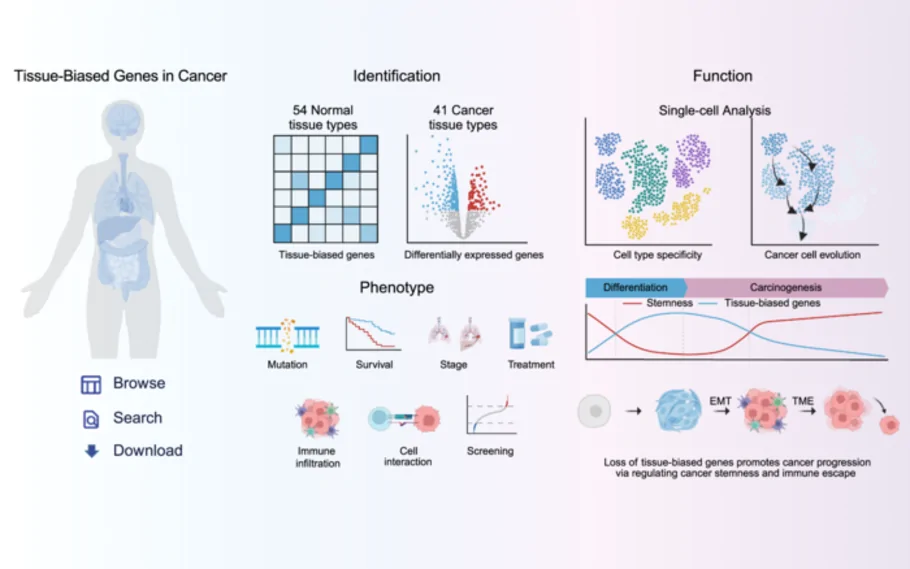

To the Editor,

Tumors are classified by tissue of origin. There are profoundly different therapy responses in different tissues. Abnormalities of tissue‐biased genes and regulons are common features among cancer types [1]. These findings led to growing attention on tissue‐biased genes and promoted the development of related databases such TissGDB [2] and GENT [3]. However, existing databases presented limited information, including the clinical relevance analysis and the changes in gene expression. It was caused by limited quantity and types of datasets and insufficient function exploration of tissue‐biased genes in previous stages. Recent advances in genetic screening and multi‐omics technologies now facilitate more comprehensive characterization of these genes [4]. Genome‐wide CRISPR screenings have identified renal lineage factor PAX8 in kidney renal cell carcinoma cell lines [5]. CRISPR can also identify immune regulators through in vivo models. Therapy sensitivity data also facilitate the discovery of tumor‐associated tissue‐biased genes, such as the C‐X‐C motif chemokine ligands (CXCLs)/C‐X‐C motif chemokine receptors (CXCRs) axis mediating chemoresistance in several cancer types [6]. Systematic multi‐omics analyses on finding common genomic features and mechanisms of tissue‐biased genes are important but lacking. Moreover, there is few single‐cell research focusing on tissue‐biased genes, which helps uncover their function on tumor evolution and immune environment. Therefore, it is urgent to build a new database to meet the research needs.

To bridge this gap, we built up the genomic and transcriptomic profiles of tissue‐biased genes across 54 normal tissues and 41 cancer types at the bulk level, and revealed their function in cancer transitions based on single‐cell RNA sequencing data. Tissue‐biased genes showed lower expression, more mutations, and higher methylation level. Single‐cell analysis found the loss of tissue‐biased genes in cancer stem cells, related to immune escape mediated by regulatory T cells (Treg). These features are common in cancer types. We further screened out four liver tissue‐biased genes highly associated with stemness and validated their function by in vitro experiments, which had potential value as therapeutic targets. Based on our findings, we developed a pan‐cancer tissue‐biased and cancer‐specific genes atlas (TBCancer). TBCancer integrates transcriptomics, proteomics, cell line therapy, CRISPR‐screening and immune‐screening data, and provides the correlation of tissue‐biased genes with cancer cell stemness and immune infiltration. Our study provided a comprehensive landscape of tissue‐biased genes in cancer and revealed that tissue‐biased genes could be essential for restricting cancer cell transition by regulating stemness and immune crosstalk.

## 1 RESULTS AND DISCUSSION

### 1.1 Pan‐cancer multi‐omics analysis reveals tissue‐biased gene patterns

**Figure 1:**
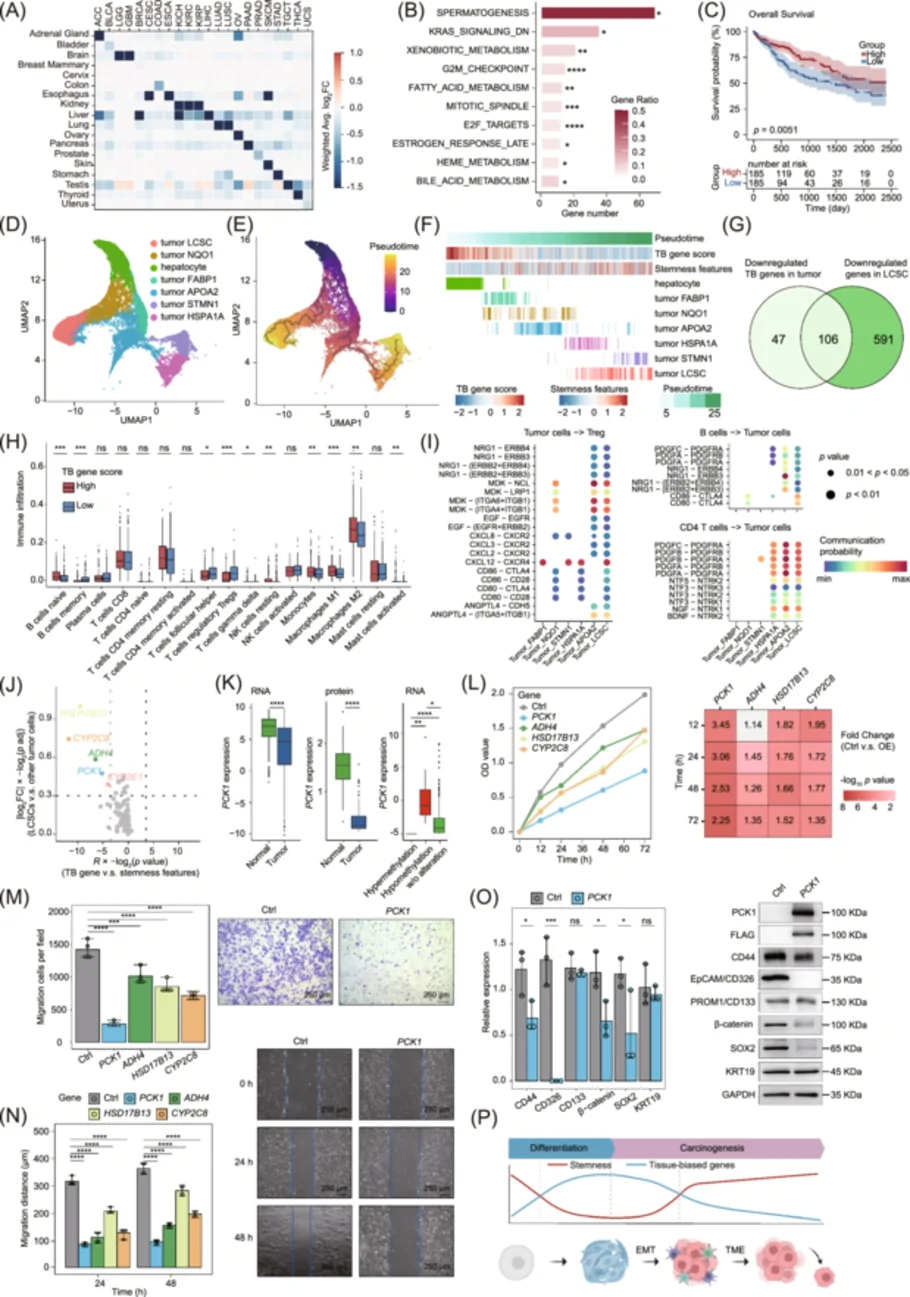
The characteristics and functions of tissue‐biased genes in cancer. (A) Heatmap of log_2_‐transformed fold changes (log_2_FC) of tissue‐biased gene expression level between normal tissue adjacent to the tumor (NAT) and tumor tissues. The abbreviations of cancer types in TCGA included adrenocortical cancer (ACC), bladder urothelial carcinoma (BLCA), brain lower grade glioma (LGG), glioblastoma multiforme (GBM), breast invasive carcinoma (BRCA), cervical and endocervical cancer (CESC), colon adenocarcinoma (COAD), esophageal carcinoma (ESCA), kidney chromophobe (KICH), kidney clear cell carcinoma (KIRC), kidney papillary cell carcinoma (KIRP), liver hepatocellular carcinoma (LIHC), lung adenocarcinoma (LUAD), lung squamous cell carcinoma (LUSC), ovarian serous cystadenocarcinoma (OV), pancreatic adenocarcinoma (PAAD), prostate adenocarcinoma (PRAD), skin cutaneous melanoma (SKCM), stomach adenocarcinoma (STAD), testicular germ cell tumor (TGCT), thyroid carcinoma (THCA), and uterine carcinosarcoma (UCS). (B) Enrichment of differentially expressed tissue‐biased in HALLMARK pathways (*p* < 0.05, hypergeometric test). (C) Stratification of TCGA LIHC cohorts with tissue‐biased gene score (median cut‐off) for overall survival (*p* < 0.05, log‐rank test). (D) Uniform Manifold Approximation and Projection (UMAP) plot of cancer cell types annotated by unique colors in liver cancer. (E) UMAP plot showing learned trajectories by monocle in cancer cell types colored by pseudotime ordering. (F) The tissue‐biased (TB) gene score and stemness score of cancer cell types at different evolutionary stages. (G) Venn diagram showing shared genes between genes significantly downregulated in LIHC tissues and genes significantly downregulated in LCSC cluster. (H) The immune infiltration fraction of liver cancer patients with high or low expression levels of liver tissue‐biased genes calculated by CIBERSORT (*p* < 0.05, two‐tailed independent *t*‐test). (I) The significant ligand‐receptor interactions between liver cancer cells and immune cells modeled by CellChat (*p* < 0.05). (J) Volcano plot showing tissue‐biased genes related to cancer cell stemness at both the bulk level and the single cell level. The *x*‐axis represented the strength of correlation between the expression level of tissue‐biased genes and stemness genes, and the vertical dashed lines indicated the thresholds for statistically significant correlation. The *y*‐axis represented the expression changes of tissue‐biased genes, and the horizontal dash lines indicated the thresholds for statistically significant changes. The *y*‐axis data were standardized to a 0–1 scale for better visual representation. Top five candidate genes and the corresponding dots were marked in different colors, and the other dots were marked in gray. (K) The expression level of *PCK1* between NAT and LIHC samples (left), and among LIHC samples with different *PCK1* methylation levels (right) (Benjamini‐Hochberg adjusted *p*‐value < 0.05, two‐tailed independent *t*‐test). (L) Cell proliferation of wild‐type Huh‐7 cells (Control, Ctrl) and Huh‐7 cells separately transfected with overexpression plasmids (OE) of *HSD17B13*, *CYP2C8*, *ADH4*, and *PCK1* measured by CCK‐8 assay. Cell migration of control and OE Huh‐7 cells measured by transwell assay (M) and scratch assay (N). (O) Western blot analysis for stemness‐related proteins in control and OE‐*PCK1* Huh‐7 cells. For the in vitro assay data (L−O), Mann–Whitney *U* tests (*p* < 0.05) were performed for pairwise comparisons between Control (*n* = 3) and OE groups (*n* = 3) at each time point. (P) Schematic diagram illustrating the interplay between progressive loss of tissue‐biased genes, elevated cancer stemness, and immune evasion in tumor evolution. The abbreviations in the diagram included epithelial‐mesenchymal transition (EMT) and tumor microenvironment (TME). Statistical significance thresholds were set at ns not significant, *p < 0.05, **p < 0.01, ***p < 0.001, and ****p < 0.0001.

In this study, we defined tissue‐biased genes as those exhibiting at least a two‐fold higher expression level in one tissue compared to all other tissues, with statistically significant differences. In diverse cancer types, tissue‐biased genes were significantly downregulated in corresponding tumors (Figure 1A, Figure S1A), which are enriched in cancer‐driving pathways including Kirsten rat sarcoma viral oncogene homolog (KRAS) and cell cycle pathways (Figure 1B). There were frequent genomic and epigenomic alterations on tissue‐biased genes, correlating with abnormal expression patterns in 5%–50% of these genes (Figure S1B). High expression of tissue‐biased genes was associated with improved overall survival (OS) and progression‐free survival (PFS) in liver cancer, kidney cancer, and lung cancer (Figure 1C, Figure S1C−E). Moreover, the patients in the late clinical and pathological stage showed decreased expression of tissue‐biased genes (Figure S1F). These results highlighted the clinical importance of tissue‐biased genes in tumors.

### 1.2 Single‐cell analysis reveals the changes and functions of tissue‐biased genes during tumor evolution

Next, we conducted single‐cell analysis to reveal the alteration and function of tissue‐biased genes during tumor evolution. To provide a clear demonstration, we chose liver hepatocellular carcinoma (LIHC) as a representative example, while our single‐cell analyses of diverse cancer types consistently supported these observations (Figures S2 and S3). Pseudotime analysis revealed two distinct evolutionary trajectories of tumor cells, both originating from normal hepatocytes. One trajectory differentiated further into the cell cluster highly expressing NAD(P)H:quinone oxidoreductase 1 (NQO1) and liver cancer stem cell (LCSC) clusters, while the other developed into cell clusters expressing fatty acid binding protein 1 (FABP1), apolipoprotein A2 (APOA2), heat shock protein family A member 1 A (HSPA1A), and stathmin 1 (STMN1) (Figures 1D,E, and S2A–C). We calculated a tissue‐biased gene score for each cell to compare the expression level of tissue‐biased genes among tumor cell clusters (see Materials and Methods). The results showed gradual inactivation of tissue‐biased genes during tumor cell evolution, especially in the LCSC cluster located at the end of the tumor evolution trajectory (Figure 1F, Figure S2D,E). Moreover, tissue‐biased gene scores were negatively correlated with tumor stemness scores across tumor cell clusters (Figure S2F) [7]. There was a considerable consistency between downregulated genes identified through bulk analysis and those detected at single‐cell resolution by comparing LCSC and other cell clusters (Figure 1G). Notably, several genes identified in our analysis, including *HP*, *TTR*, *APOH*, *HPX*, and *SAA1*, have been previously implicated in the regulation of tumor stemness [8] (Table S1). Acquisition of stemness is associated with the oncogenic dedifferentiation. Tissue‐biased genes showed activation during embryo development but silencing in cancer progression, implicating their functional loss facilitated epithelial transformation and stemness acquisition (Figure S2G−H).

Additionally, our analysis revealed that tissue‐biased genes participate in immune regulation. Immune infiltration analysis showed that patients with lower tissue‐biased gene expression have decreased infiltration of B cell and macrophage, and increased infiltration of Treg (Figure 1H). Single‐cell analysis showed more receptor‐ligand pairs between LCSC and Treg compared to other cancer cell clusters, which included midkine (MDK), nucleolin (NCL), cytotoxic T‐lymphocyte associated protein 4 (CTLA4), and CXCR known to affect Treg proliferation and maturation [9] (Figure 1I). We also observed activated receptor‐ligand pairs between LCSC and other immune cells, including B cell, CD4 + T cell, M1 macrophage, and M2 macrophage (Figure S4). These results indicated that the loss of tissue‐biased genes might impair the immune surveillance on LCSC. Pioneering investigations revealed that the crosstalk between tumor stemness pathway and immune response modulated cancer progression. Our results indicated that tissue‐biased genes might participate in these mechanisms.

Herein, we supposed that a subset of epithelial cells may lose function of tissue‐biased genes, gain stem cell‐like features, and escape immune surveillance, contributing to cancer cell proliferation and metastasis. To screen for tissue‐biased genes that are highly correlated with stemness, we calculated the correlation between the expression of tissue‐biased genes and stemness genes at both bulk and single‐cell level datasets (Figure 1J, Table S2). Based on our selection criteria, four top candidate genes were identified for further analysis and experimental validation. In The Cancer Genome Atlas (TCGA) LIHC cohort, *PCK1*, *ADH4*, *HSD17B13*, and *CYP2C8* were significantly downregulated in tumor and could be affected by DNA methylation and copy number variation (CNV) alteration (Figure 1K, Figure S5). These were common features of tissue‐biased genes associated with tumor. Experiments showed that individual overexpression of *PCK1*, *ADH4*, *HSD17B13*, and *CYP2C8* decreased the proliferation ability and migration ability of liver cancer cells (Figure 1L−N, Figure S5). Furthermore, we observed decreased expression of stemness genes in tumor cells transfected with *ADH4* or *PCK1* plasmid (Figure 1O). These results further validated the roles of tissue‐biased genes in tumor evolution (Figure 1P).

### 1.3 TBCancer: A comprehensive database for tissue‐biased and cancer‐specific genes

**Figure 2:**
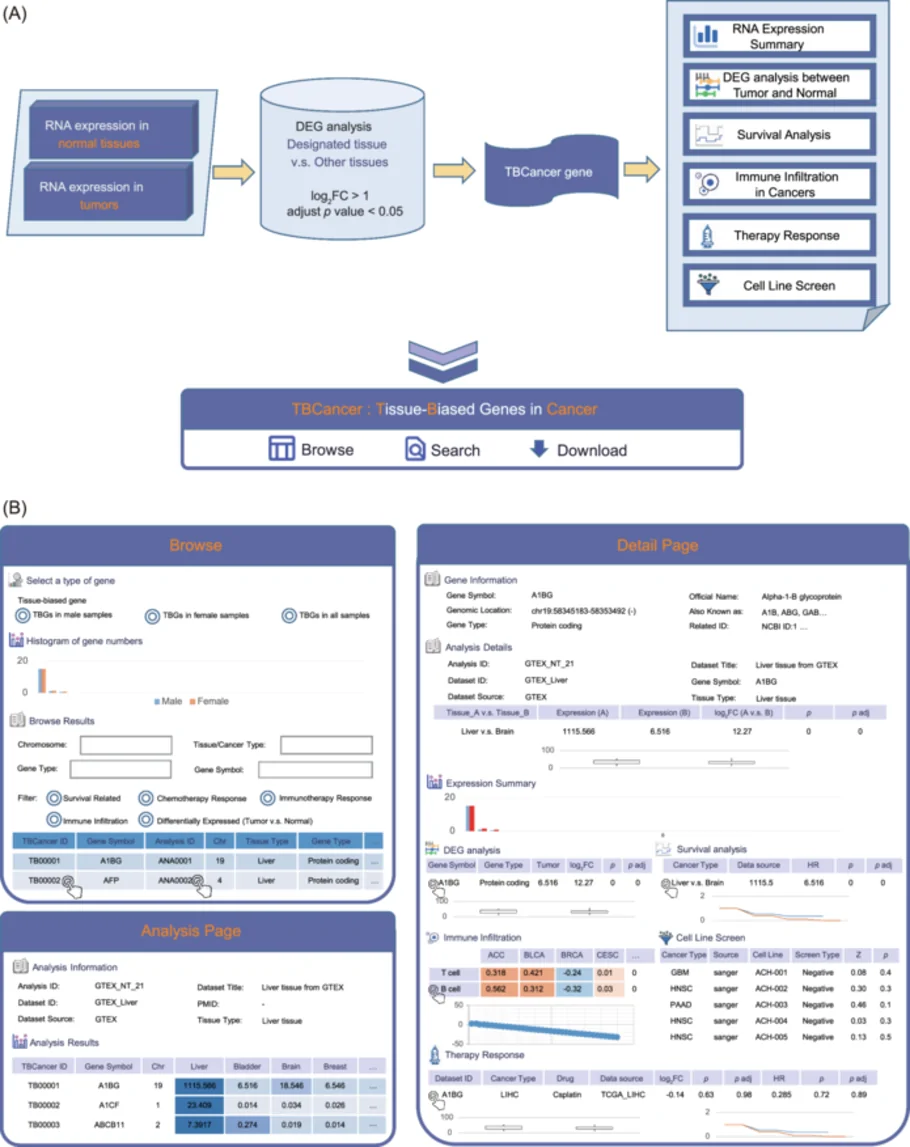
Overall design and construction of TBCancer. (A) The construction pipeline and structure of TBCancer database. (B) The browse and detail pages including six analysis modules in TBCancer database.

Based on the above exploration, we developed a pan‐caner tissue‐biased and cancer‐specific genes atlas (TBCancer) for better understanding on tissue‐biased genes and for screening candidate biomarkers and therapeutic targets. TBCancer can be accessed at http://tsbcancer.canceromics.org/. More than 10,000 tissue‐biased genes from 54 normal tissues were included in TBCancer. Their features and functions in cancers were analyzed by integrating multi‐omics data and clinical information of the large‐scale pan‐cancer cohorts from TCGA [10] and Gene Expression Omnibus (GEO) repository [11] (Figure 2A,B).

The Browser page provides the data source information and an overview of tissue‐biased genes from diverse cancer types. Users can select specific tissue/cancer types and available data types to generate a gene list of interest, then click the database ID to obtain detailed gene information. Alternatively, users can directly search for genes of interest on the Search page. The detailed page provides basic gene information, gene related pathways, and six analysis modules (Figure 2A,B, Figure S6). Expression summary visualized gene expression specificity across normal and tumor tissue types. Differential gene expression analysis revealed the changes of tissue‐biased genes across cancer types, with the downregulation of genes in their matched cancers being particularly noteworthy. These tissue‐biased and cancer‐specific genes deserved further analysis. Survival analysis was performed using the Kaplan‐Meier method based on clinical information (OS and PFS) of TCGA cohort datasets. Based on our analysis, we highlighted the importance of tissue‐biased genes in immune regulation. In TBCancer, the correlation between gene and immune infiltration in cancers was presented through four methods including ssGSEA, CIBERSORT, TIMER, and MCP counter. This module helps to find out immune‐related genes and their interactions with immune cells. Major advantages offered by TBCancer are plenty of therapy sensitivity data and CRISPR screening data, which are limited in other databases. It displayed the correlation between gene and chemotherapy and the correlation between gene and immunotherapy, through comparing the gene expression level and survival time between responder and nonresponder patients. The gene expression level at cell lines treated with drug at half‐maximal response dose (IC_50_) also provided clues to the mechanisms of tissue‐biased genes. Additionally, we gathered CRISPR screening datasets from the Cancer Cell Line Encyclopedia [12], Catalogue Of Somatic Mutations In Cancer [13], and GEO [11]. This massively parallel analysis helps to find out genes affecting cancer cell proliferation and immune cell phenotype.

Integrative analysis using TBCancer helps to identify key cancer‐associated tissue‐biased genes, with functional insights provided by single‐cell immune profiling through our TIGER database resource [14]. Taking *HSD17B13* as an example, TBCancer showed its specificity in liver tissue, downregulation in liver cancer, and the association between higher expression and better prognosis. Treatment with Sorafenib showed increased expression of *HSD17B13*, and the negative correlation between *HSD17B13* expression and MP470 IC_50_ implied that this gene may serve as a biomarker for enhanced drug sensitivity. Additionally, its expression is positively correlated with neutrophil infiltration. Single‐cell analysis through TIGER showed its enrichment in tumor cells and its loss in evolutionary trajectories, and more cell–cell interactions between tumor cells and myeloid cells. The co‐expression analysis helps to find out related genes to tissue‐biased genes of interest, such as stemness genes and immune genes we have described and other important genes in cancer.

## 2 CONCLUSION

In this study, we characterized common features and mechanisms of tissue‐biased genes in cancer through systematic multi‐omics analyses: (1) tissue‐biased genes were significantly decreased in cancer, a phenomenon at least partially driven by CNV and DNA methylation; (2) the distribution and alteration of tissue‐biased genes were specific in epithelial cells and cancer cells; (3) the loss of tissue‐biased genes might function on cancer cell stemness and immune escape during tumorigenesis. Based on our findings and to address current database limitations, we developed the TBCancer database, providing the users with visual interface, succinct and effective modules, and easy operation.

These findings not only highlight the underappreciated roles of tissue‐biased genes in tumor biology but also establish TBCancer as an essential resource for the emerging field.

## 3 METHODS

Detailed procedures for experimental methods, data analysis and statistical methodology, and TBCancer database construction are available in the Supplementary Information.

## AUTHOR CONTRIBUTIONS

**Zhuobin Lin**: Methodology; visualization; project administration; writing—original draft. **Kunhua Hu**: Funding acquisition; project administration; methodology; writing—original draft. **Hongyan Sun**: Validation; methodology; project administration. **Xiaoqiong Bao**: Project administration; software. **Lin Tang**: Project administration; software. **Wei Liu**: Supervision; conceptualization; funding acquisition; writing—review and editing. **Zhixiang Zuo**: Conceptualization; supervision; funding acquisition; writing—review and editing. **Zhihang Chen**: Conceptualization; supervision; writing—review and editing; validation; visualization.

## CONFLICT OF INTEREST STATEMENT

The authors declare no conflicts of interest.

## ETHICS STATEMENT

No animals or humans were involved in this study.

## Supporting information


**Figure S1.** Pan‐cancer profiles of tissue‐biased genes in tumors.
**Figure S2.** Single‐cell analysis revealed dramatic loss of tissue‐biased genes during tumor evolution in LIHC.
**Figure S3.** Single‐cell analysis on tissue‐biased genes in NSCLC, KIRP, and CRC.
**Figure S4.** Abnormal expression of tissue‐biased genes affects immune crosstalk in tumor.
**Figure S5.** The inactivation of tissue‐biased genes in liver cancer enhances tumor stemness and affects tumor proliferation and metastatic ability.
**Figure S6.** The function and usage of TBCancer.

**Table S1.** Intersection of genes downregulated in LCSC relative to hepatocyte and tissue‐biased genes downregulated in liver tumor relative to normal tissue adjacent to the tumor.
**Table S2.** List of liver‐biased genes highly associated with cancer cell stemness.
**Table S3.** The reference gene set for tumor cell stemness scores.
**Table S4.** List of the primers used in this study.
**Table S5.** List of the data resources that are integrated by TBCancer.

## Data Availability

The data that support the findings of this study are openly available in UCSC Xena at https://xena.ucsc.edu/, reference number 10. The data that supports the findings of this study are publicly available and obtained from open‐access sources. The code scripts of this study are available at https://github.com/zhuobinlin/TBCancer. Supplementary materials (methods, figures, tables, graphical abstract, data sources, slides, videos, Chinese translated version, and update materials) may be found in the online DOI or iMetaOmics http://www.imeta.science/imetaomics/.
