# Supplementary material for: TBCancer: A database for exploring characteristics and functions of tissue‐biased genes in cancer
Source: IMetaOmics. 2025 Jun 29;2(4):e70039. doi: 10.1002/imo2.70039 (PMC12806124; doi:10.1002/imo2.70039)
Supplement: Supplementary file 1 — Figure S1. Pan‐cancer profiles of tissue‐biased genes in tumors. Figure S2. Single‐cell analysis revealed dramatic loss of tissue‐biased genes during tumor evolution in LIHC. Figure S3. Single‐cell analysis on tissue‐biased genes in NSCLC, KIRP, and CRC. Figure S4. Abnormal expression of tissue‐biased genes affects immune crosstalk in tumor. Figure S5. The inactivation of tissue‐biased genes in liver cancer enhances tumor stemness and affects tumor proliferation and metastatic ability. Figure S6. The function and usage of TBCancer. [file IMO2-2-e70039-s001.docx]

**Supporting information to**

**TBCancer: A database for exploring** **characteristics and functions of tissue-biased genes in cancer**

**Running title:** TBCancer: A database for tissue-biased genes in cancer

Zhuobin Lin^1,#^, Kunhua Hu^1,#^, Hongyan Sun^2,#^, Xiaoqiong Bao^2,3,#^, Lin Tang^3^, Wei Liu^1,*^, Zhixiang Zuo^2,3,*^, and Zhihang Chen^2,4,*^

^1^Guangdong Key Laboratory of Liver Disease Research, The Third Affiliated Hospital of Sun Yat‑sen University, Sun Yat-sen University, Guangzhou, China

^2^State Key Laboratory of Oncology in South China, Cancer Center, Collaborative Innovation Center for Cancer Medicine, Sun Yat-sen University, Guangzhou, China

^3^School of Life Sciences, Sun Yat-sen University, Guangzhou, China

^4^Department of Interventional Medicine, The Fifth Affiliated Hospital of Sun Yat-sen University, Zhuhai, China

^#^These authors contributed equally: Zhuobin Lin, Kunhua Hu, Hongyan Sun, and Xiaoqiong Bao.

^*^Correspondence: [chenzhihang532491@163.com](mailto:chenzhihang532491@163.com) (Zhihang Chen), [zuozhx@sysucc.org.cn](mailto:zuozhx@sysucc.org.cn) (Zhixiang Zuo), and [lwei6@mail.sysu.edu.cn](mailto:lwei6@mail.sysu.edu.cn) (Wei Liu)

**Supplementary Notes**

**Methods**

**Identification and annotation of tissue-biased genes**

In this study, we utilized the normal tissue transcriptomic sequencing data from the Genotype-Tissue Expression (GTEx) [1] for analysis. We ultilized the ‘limma’ (v3.54.2) [2] package to perform the pairwise differential analysis between tissues, and defined tissue-biased genes if they met the criteria: (1) significant differential expression, specifically, adjusted *p*-values (*p* adj) < 0.05 by Benjamini-Hochberg (BH) correction, and (2) log_2_ fold-change (log_2_FC) > 1.00 relative to all other tissues. These genes were further used for building the database and subsequent analyses.

In this study, we used the UCSC Toil RNAseq Recompute Compendium datasets [3] for downstream analysis. It is worth noting that the batch effects and technical bias between The Cancer Genome Atlas (TCGA) [4] datasets have been well reduced through the recomputing procedures and consistent meta-analysis. Differential gene expression analysis between normal and tumor were performed to screen out tissue-biased genes associated with cancer. The confounding factors might have effect on gene expression, such as clinical information and heterogeneity. Here, we selected matched normal tissue adjacent to the tumor (NAT) and tumor tissues. Differential expression analysis was performed using ‘limma’ (v3.54.2) [2] with paired statistical tests, incorporating linear models that adjusted for key clinical covariates including treatment history and pathological stage.

**Definition and calculation of tissue-biased gene score and stemness score**

We assessed the global expression level of tissue-biased genes in each tumor patient using single sample gene set enrichment analysis (ssGSEA) in the ‘GSVA’ package (v2.0.7) [5] and named the indicator the tissue-biased (TB) gene score. We constructed a reference gene set related to cell stemness (Table S3) form previous studies and calculated the ssGSEA scores as indicator of overall expression level of stemness genes. Similarly at the single-cell level, we separately calculated the TB gene score and the stemness score of every single cell by ssGSEA and project them to different cell clusters.

To find out tissue-biased genes correlated with stemness, we performed correlation analysis between gene expression level and stemness level at bulk level by Pearson’s correlation method, and calculated the $R\times-{log}_{2}(p \mathrm{value})$ as the indicator of the strength of correlation. We performed differentially expressed genes (DEGs) analysis at single-cell level using Seurat (v4.3.0) [6] "FindAllMarkers" function to perform a Wilcoxon rank-sum test, and calculated the $\left| {log}_{2}\mathrm{FC} \right|\times-{log}_{2}(p \mathrm{adj})$ as the indicator of expression changes of tissue-biased genes.

**Genomic alterations on tissue-biased genes**

Genetic data examined in this study was from TCGA cohorts [4] with paired gene expression data and available clinical information. The single nucleotide variant (SNV) results of MuTect2 [3, 7] were used, which included the mutated genes and their variant types of each sample. We divided cancer samples into SNV and No-SNV group based on whether SNVs were detected on the target gene. We compared the gene expression level between groups using independent *t*.test method. Gene expression was considered to be affected by SNV if there were statistically significant differences across groups (*p* adj < 0.05). Due to the diversity of SNV types and their varying impacts on gene expression, we did not mandate consistent expression changes (either up- or down-regulation) relative to the No-SNV baseline.

For the copy number variation (CNV) results of GISTIC [3, 8], we firstly converted gene Ensemble ID to gene symbol based on the cross-references from TCGA [4]. There were originally 19,729 Ensemble IDs and 19,662 gene symbols after conversion. In the result, ‘1’ value means an amplifying number of DNA segments (amplification), ‘-1’ value means a decreasing number of DNA segments (deletion), and ‘0’ value means no CNV detected in a specific gene. We divided cancer samples into the Amplification, Deletion, and No-CNV group based on the CNV alteration of the target gene. Independent *t*-test on gene expression was performed between: 1) Amplification and Deletion, 2) Amplification and No-CNV, and 3) Deletion and No-CNV groups. Gene expression was considered to be affected by CNV if it met the criteria: (1) statistically significant differences across groups (*p* adj < 0.05), and (2) a consistent expression change pattern (Amplification group > No-CNV group > Deletion group).

**Epigenomic alterations on tissue-biased genes**

Illumina Infinium HumanMethylation450 datasets were available in TCGA [4]. The *β*-value, defined as the ratio of the methylated signal over the total signal, is the indicator of DNA methylation level. Consistent with previous studies, sites are considered hypermethylated with *β*-value > 0.6, sites are considered hypomethylated with *β*-value < 0.4, and other sites are in intermediate methylation statuses. There were near half a million probes and a probe might be corresponding to more than one gene. Based on the ID/Gene mapping files from TCGA [4], we assigned probes to genes, and for genes with multiple sites or probes, we calculated the mean of *β*-values. After this, each gene had a unique *β*-value and then classified as hypermethylated, hypomethylated or intermediate in every sample. We divided cancer samples into the Hypermethylation, Hypomethylation, and Normal-Methylation group based on the *β*-value of the target gene. Independent *t*-test on gene expression was performed between: 1) Hypermethylation and Hypomethylation, 2) Hypermethylation and Normal-Methylation, and 3) Hypomethylation and Normal-Methylation groups. Gene expression was considered to be affected by DNA methylation if it met the criteria: (1) statistically significant differences across groups (*p* adj < 0.05), and (2) a consistent expression change pattern (Hypomethylation group > Normal-Methylation group > Hypermethylation group).

A target gene in a specific cancer might be affected by SNV, CNV, DNA methylation, or their combined effects. We systematically analyzed their individual impacts on gene expression and counted the number of affected genes.

**Tissue-biased gene expression and survival analysis**

In TCGA datasets [4], patients with available RNA-seq data and survival data were used for survival analysis. Patients were classified into high-expression group (High) and low-expression group (Low) based on TB gene score. We utilized the ‘survival’ package (v3.5.5) [9] in R to determine the survival duration and status of patients in the two groups. To find out the clinical-related genes, we performed Kaplan-Meier curves, log-rank test, and Cox proportional hazards regression on all genes in all cancer types.

**Pathway enrichment analysis**

We performed GSEA enrichment analysis using the HALLMARK [10] tumor feature gene set as reference. We divided cancer samples into high-expression group and low-expression group based on TB gene score or the expression level of single gene. All genes were ranked based on differential analysis between two groups (log_2_FC and *p* adj, limma (v3.54.2) [2] ), and then were used for GSEA. A positive enrichment score meant the activation of pathway, while a negative enrichment score meant the inactivation of pathway in the low-expression group.

We performed hypergeometric test on the differentially expressed tissue-biased genes between normal tissue and tumor and HALLMARK [10] genes of different pathways. A *p* adj < 0.05 indicated tissue-biased genes enriched in the HALLMARK [10] pathway.

**Preprocessing of single-cell transcriptomic sequencing data and discrimination of malignant and non-malignant cells**

We obtained raw FASTQ files from single-cell RNA sequencing (scRNA-seq) data across multiple cancer types. Cell Ranger (v6.1.1) [11] was utilized for quantifying the single-cell transcriptome to obtain the expression matrix. We filtered out low-quality cells based on these conditions: (1) the proportion of mitochondrial genes > 15 %, (2) the number of mitochondrial genes < 300 or > 3,000, and (3) unclassified cells that express more than three types of immune markers. The ‘NormalizeData’ function in Seurat (v4.3.0) [6] was used for data normalization. Subsequently, Seurat (v4.3.0) [6] was employed for cell cycle assignment. ‘FindVariableFeatures’ function was used to select genes required for subsequent analyses. Principal Component Analysis (PCA) was used for dimensionality reduction and denoising of single-cell transcriptomic data. ‘FindNeighbors’ function in Seurat (v4.3.0) [6] was then applied for *k*-means clustering of the single-cell transcriptomic data. Finally, the ‘FindAllMarkers’ parameter was used to conduct Wilcoxon rank-sum tests to identify genes with significantly higher expression in specific cell types. The highly expressed genes in each cell cluster were considered as feature genes for the annotation of cell types. Subsequently, Uniform Manifold Approximation and Projection (UMAP) was used for dimensionality reduction and visualization of the single-cell transcriptomic data. To discriminate malignant and non-malignant cells, the ‘inferCNV’ (v1.3.3) [12] function was used to infer large-scale CNV in the single-cell transcriptomic data. Differences between each cell cluster and normal epithelial cells were observed from the copy number variation map. Non-malignant cells typically showed no apparent CNV patterns, while malignant tumor cells exhibited frequent copy number variations. To verify the classification, we further evaluated the expression of malignant tumor-specific and normal epithelial-specific marker genes within the malignant and non-malignant cell populations.

**Tumor cell subtype pseudotime and cell-cell communication analysis**

To understand the differentiation trajectories of different tumor cell subgroups, we performed trajectory analysis using the pseudotime inference algorithm Monocle 3 (v1.0.0) [13]. In this study, we utilized ‘CellChat’ (v1.4.0) [14] package in R to conduct an analysis of cell-cell communication. Only the ligand-receptor interactions meeting the threshold of *p* value < 0.05 were retained for further analysis.

**Cell culture and transfections**

Human hepatoma Huh-7 cell line were purchased from the Cell Bank of Type Culture Collection (Shanghai, China). As described in previous work, cells were grown in the RPMI-1640 medium (Invitrogen) supplemented with 10% FBS (Gibco) and 1% penicillin/streptomycin (Invitrogen) at 5% CO_2_ and 37 ℃. The medium was changed every two to three days.

The complete open reading frames (ORFs) sequence of *HSD17B13*, *ADH4*, *PCK1* and *CYP2C8* were retrieved from NCBI Gene. Each of these sequences was separately cloned into a plasmid vector pcDNA3.1-3×Flag (Thermo Fisher Scientific). The sequences of primers were listed in Table S4. Cells were transiently transfected with one of the recombinant plasmids using Lipofectamine™ 3000 reagent (Invitrogen) according to the manufacturer’s instructions. Cells transfected with empty vectors were used as a control. At 8 hours post-transfection, we replaced medium with fresh RPMI-1640 medium. Transfected cells were used for the following experiments after 0, 12, 24, 48, and 72 hours of culturing. The transduction efficacy was verified by GFP expression as determined by fluorescence microscopy, and target gene expression by qPCR and western blotting.

**Western blot**

Proteins were extracted from the samples using a Proteins Extraction Kit (CWBIO) and quantified with BCA-Reagents (CWBIO). Proteins were separated by SDS-PAGE on 10% SDS-PAGE and then electroblotted onto polyvinylidene fluoride membranes (PVDF) (Millipore). The membranes were blocked with 5% skim milk in PBS at room temperature (RT) for 1 hour, then incubated overnight at 4 ℃ with the following primary antibodies: Anti-HSD17B13 antibody (Abcam ab122036 1:1000); Anti-ADH4 antibody (Poteintech 16474-1-AP 1:500); Anti-PCK1 antibody (Poteintech 16754-1-AP 1:500); Anti-CYP2C8 antibody (Poteintech 16546-1-AP 1:500); Anti-SOX2 antibody (Poteintech 20118-1-AP 1:500); Anti-PRDM1 antibody (Cell Signaling Technology #9115 1:1000); Anti-CD44 antibody (Poteintech 15675-1-AP 1:500); Anti-KRT19 antibody (Poteintech 10712-1-AP 1:500); Anti-EpCAM antibody (Poteintech 21050-1-AP 1:500).

After washing the membranes three times with PBS-T and incubation with an HRP-linked secondary antibody for 1 hour at RT, the blotting signal was visualized with enhanced chemiluminescence immunoblotting detection reagents (Immobilon Crescendo Western HRP Substrate). Densitometric values of the Western blot bands were analyzed by Image Lab Software (BIO-RAD, USA), semiquantified based on signal intensity, and normalized to GAPDH expression.

**Cell viability assay**

Cell Counting Kit-8 (CCK-8; Beyotime) was applied for detecting cell viability following the manufacturer’s instructions. Cells were seeded in 96-well plates at a density of 600 cells/well. Wells containing 100 μL medium alone (without cells) were used as blank controls. CCK-8 assays were performed at the 12, 24, 48, and 72 hours of post-seeding. Absorbance, also known as optical density (OD), at 450 nm was measured using a microplate reader (Bio-Rad). The blank control served as baseline.

**Cell migration and invasion assay**

Cell migration of Huh-7 cells was evaluated using a transwell assay and scratch test. Transwell chambers (8-μm pore size; Corning) were used. In 24-well culture plates, 500 μL RPMI-1640 with 10% FBS was added into each lower chamber. Cells (1×10^4^ cells/well) in 200 μL RPMI-1640 without FBS were seeded into each upper chamber. After incubation for 24 hours, cells adhered to the lower surface were washed with PBS, fixed with methanol, and stained with 1% crystal violet. Invaded cells were photographed and counted in 5 different fields of view using an optical microscope.

For the scratch assay, cells were seeded in 6-well plates at 90% confluency. After incubation for 24 hours, an even scratch wound was made using a disposable pipette, followed by washing with PBS. Cells were then incubated in supplemented medium and immediately imaged for the first time point (0 hour), and then subsequently imaged at 24 and 48 h. Cell migration was calculated by the scratch distance.

**Statistical analysis**

In addition to the aforementioned analytical approaches, we chose appropriate methods based on data characteristics. For pairwise comparisons, the large datasets with normal distribution evaluated by Shapiro-Wilk tests, such as the TCGA cohorts [4], were examined using independent two-sample *t*-tests. The small datasets or non-normally distributed data, such as the *in vitro* experiments with three replicates, were analyzed by Mann-Whitney *U* tests. All statistical tests were two-tailed with *α* = 0.05. For multiple testing, we applied the Benjamini-Hochberg method to control false discovery rate at *α* = 0.05 and to calculate the adjusted *p*-values (*p* adj). All correlation analyses were conducted using Pearson’s correlation coefficient to evaluate linear relationships between continuous variables.

**Note 2. Database constructions**

**Data sources**

The data used in the database was collected from TCGA dataset available in UCSC Xena [3, 4]. The dataset consists of aligned and quantified tissue RNA-seq data, including 11,072 cancer tissues and matched adjacent normal samples across 33 cancer types. Additionally, tumor and normal tissue RNA-seq and gene chip data along with clinical information, were collected from the Gene Expression Omnibus (GEO) repository [15], yielding a total of 6,155 samples. After integrating these data with the TCGA RNA-seq data [4], a total of 17,277 samples from 41 cancer types were included. Gene expression data from 7,862 samples of 54 normal tissue types from the GTEx project were collected [1]. A total of 2,496 protein mass spectrometry samples with patient information from 14 cancer types were collected from the Clinical Proteomic Tumor Analysis Consortium (CPTAC) [16]. Single-cell sequencing data and clinical information were retrieved and collected from various databases, including GEO [15], Genome Sequence Archive (GSA) [17], and the European Bioinformatics Institute (EMBL-EBI) [18]. Fourteen single-cell sequencing datasets were included in this study, totaling 265,648 epithelial cells and malignant cells. RNA-seq data from tumors with chemotherapy information were collected from the TCGA database [4], GEO database[15], and Sequence Read Archive (SRA) [19]. A total of 4,075 samples from 91 tumor chemotherapy-related gene expression sequencing runs were included. Additionally, gene expression data from 25 tumors and 1,471 samples treated with immunotherapy were collected from the GEO [15] and SRA [19] databases. Data on half-maximal inhibitory concentrations of 251 chemotherapy, targeted therapy, and immunotherapy drugs across 609 cell lines were downloaded from the Genomics of Drug Sensitivity in Cancer (GDSC) [20] database for integrated analysis. CRISPR-screening data from 791 tumor cell lines of 30 cancer types and 31 immune-related CRISPR-screening datasets were collected by searching the Cancer Cell Line Encyclopedia (CCLE) [21], Catalogue Of Somatic Mutations In Cancer (COSMIC) [22], GEO database [15], and PubMed [23]. For detailed descriptions and statistical information of the aforementioned data resources, please refer to Table S5.

**Quality control and pre-processing**

Preprocessed transcriptomic data from TCGA [4] and GTEx [1] datasets were used for tumor and normal tissue analysis. For other transcriptomic sequencing data retrieved from databases such as GEO [15], the provided sample identifiers such as SRA accession numbers or GSE numbers were used to download the raw data. The raw data downloaded from GEO were in SRA format, and were converted into fastq format by Sra-tools (v2.11.0) [19]. Initially, FastQC (v0.11.9) [24] was employed to perform quality control on the transcriptomic data. We assessed sequence duplication, adapter content, GC bias, nucleotide composition, and base call quality to evaluate the overall library quality. Samples with errors exceeding 30% after quality control assessment were excluded in further analysis. Subsequently, the STAR (v2.7.10) [25] was used to align the transcriptomic data to the current version of human reference genome (GRCh38). Finally, featureCounts (v2.0.2) [26] was used for gene expression quantification and generated a gene count matrix. The transcripts per kilobase million (TPM) metric was used for normalization, facilitating relative comparisons of gene expression levels among different samples for subsequent analysis. DNA microarray data were downloaded using the GSE numbers provided by the GEO database [15]. Subsequently, gene annotations were integrated from Ensembl [27] and RefSeq [28] databases. Probe sequences from different platforms were aligned to the human genome, and annotated to coding genes based on coordinates. Different strategies were used for normalization of expression datasets from different platforms. For expression data from Affymetrix, we tried to obtain the raw CEL files, and used the Robust Multi-array Average (RMA) algorithm (v1.56.0) [29] for standardization. For data from Agilent, Illumina, and other platforms, the ‘limma’ package (v3.54.2) [2] was used for computation and normalization. For studies lacking raw data, the data matrices provided in the GEO [15] database were used directly. All data were normalized at the probe level and log_2_-transformed. Finally, we mapped probes to gene names based on the probe annotation file.

**Database construction**

All data in TBCancer were stored and managed using MySQL [30] tables. The server-side development was based on Java, while the web interface was implemented using HyperText Markup Language (HTML) [31], Cascading Style Sheets (CSS) [31, 32], and JavaScript (JS) [33]. To present data more efficiently and intuitively, the website incorporated various interactive charts and graphs. Interactive heatmap displays of expression abundance and differential expression were constructed using the Ant Design toolkit [34], while box plots illustrating differential expression were generated using Echarts [35].

**References**

1. The GTEx Consortium, François Aguet, Shankara Anand, Kristin G. Ardlie, Stacey Gabriel, Gad A. Getz, Aaron Graubert, et al. 2020. “The GTEx Consortium atlas of genetic regulatory effects across human tissues.” *Science* 369: 1318−1330. <https://doi.org/10.1126/science.aaz1776>

2. Ritchie, Matthew E., Belinda Phipson, Di Wu, Yifang Hu, Charity W. Law, Wei Shi, Gordon K. Smyth. 2015. “Limma powers differential expression analyses for RNA-sequencing and microarray studies.” *Nucleic Acids Research* 43: e47−e47. <https://doi.org/10.1093/nar/gkv007>

3. Vivian, John, Arjun Arkal Rao, Frank Austin Nothaft, Christopher Ketchum, Joel Armstrong, Adam Novak, Jacob Pfeil, et al. 2017. “Toil enables reproducible, open source, big biomedical data analyses.” *Nature Biotechnology* 35: 314−316. <https://doi.org/10.1038/nbt.3772>

4. The Cancer Genome Atlas Research Network, John N Weinstein, Eric A Collisson, Gordon B Mills, Kenna R Mills Shaw, Brad A Ozenberger, Kyle Ellrott, Ilya Shmulevich, Chris Sander, Joshua M Stuart. 2013. “The Cancer Genome Atlas Pan-Cancer analysis project.” *Nature Genetics* 45: 1113−1120. <https://doi.org/10.1038/ng.2764>

5. Hänzelmann, Sonja, Robert Castelo, Justin Guinney. 2013. “GSVA: gene set variation analysis for microarray and RNA-Seq data.” *BMC Bioinformatics* 14: 7. <https://doi.org/10.1186/1471-2105-14-7>

6. Hao, Yuhan, Tim Stuart, Madeline H. Kowalski, Saket Choudhary, Paul Hoffman, Austin Hartman, Avi Srivastava, et al. 2024. “Dictionary learning for integrative, multimodal and scalable single-cell analysis.” *Nature Biotechnology* 42: 293−304. <https://doi.org/10.1038/s41587-023-01767-y>

7. Cibulskis, Kristian, Michael S. Lawrence, Scott L. Carter, Andrey Sivachenko, David Jaffe, Carrie Sougnez, Stacey Gabriel, Matthew Meyerson, Eric S. Lander, Gad Getz. 2013. “Sensitive detection of somatic point mutations in impure and heterogeneous cancer samples.” *Nature Biotechnology* 31: 213−219. <https://doi.org/10.1038/nbt.2514>

8. Beroukhim, Rameen, Gad Getz, Leia Nghiemphu, Jordi Barretina, Teli Hsueh, David Linhart, Igor Vivanco, et al. 2007. “Assessing the significance of chromosomal aberrations in cancer: methodology and application to glioma.” *Proceedings of the National Academy of Sciences* 104: 20007−20012. <https://doi.org/10.1073/pnas.0710052104>

9. Therneau, Terry M., Patricia M. Grambsch. 2000. “The Cox model.” *Modeling survival data: extending the Cox model*, Springer New York, 39−77. <https://doi.org/10.1007/978-1-4757-3294-8_3>

10. Liberzon, Arthur, Chet Birger, Helga Thorvaldsdóttir, Mahmoud Ghandi, Jill P Mesirov, Pablo Tamayo. 2015. “The Molecular Signatures Database hallmark gene set collection.” *Cell Systems* 1: 417−425. <https://doi.org/10.1016/j.cels.2015.12.004>

11. Zheng, Grace X. Y., Jessica M. Terry, Phillip Belgrader, Paul Ryvkin, Zachary W. Bent, Ryan Wilson, Solongo B. Ziraldo, et al. 2017. “Massively parallel digital transcriptional profiling of single cells.” *Nature Communications* 8: 14049. <https://doi.org/10.1038/ncomms14049>

12. Patel, Anoop P., Itay Tirosh, John J. Trombetta, Alex K. Shalek, Shawn M. Gillespie, Hiroaki Wakimoto, Daniel P. Cahill, et al. 2014. “Single-cell RNA-seq highlights intratumoral heterogeneity in primary glioblastoma.” *Science* 344: 1396−1401. <https://doi.org/10.1126/science.1254257>

13. Trapnell, Cole, Davide Cacchiarelli, Jonna Grimsby, Prapti Pokharel, Shuqiang Li, Michael Morse, Niall J. Lennon, Kenneth J. Livak, Tarjei S. Mikkelsen, John L. Rinn. 2014. “The dynamics and regulators of cell fate decisions are revealed by pseudotemporal ordering of single cells.” *Nature Biotechnology* 32: 381−386. <https://doi.org/10.1038/nbt.2859>

14. Jin, Suoqin, Christian F. Guerrero-Juarez, Lihua Zhang, Ivan Chang, Raul Ramos, Chen-Hsiang Kuan, Peggy Myung, Maksim V. Plikus, Qing Nie. 2021. “Inference and analysis of cell-cell communication using CellChat.” *Nature Communications* 12: 1088. <https://doi.org/10.1038/s41467-021-21246-9>

15. Barrett, Tanya, Stephen E Wilhite, Pierre Ledoux, Carlos Evangelista, Irene F Kim, Maxim Tomashevsky, Kimberly A Marshall, et al. 2013. “NCBI GEO: archive for functional genomics data sets--update.” *Nucleic Acids Research* 41: D991−D995. <https://doi.org/10.1093/nar/gks1193>

16. Edwards, Nathan J., Mauricio Oberti, Ratna R. Thangudu, Shuang Cai, Peter B. McGarvey, Shine Jacob, Subha Madhavan, Karen A. Ketchum. 2015. “The CPTAC Data Portal: a resource for cancer proteomics research.” *Journal of Proteome Research* 14: 2707−2713. <https://doi.org/10.1021/pr501254j>

17. Wang, Yanqing, Fuhai Song, Junwei Zhu, Sisi Zhang, Yadong Yang, Tingting Chen, Bixia Tang, et al. 2017. “GSA: Genome Sequence Archive.” *Genomics, Proteomics & Bioinformatics* 15: 14−18. <https://doi.org/10.1016/j.gpb.2017.01.001>

18. Thakur, Matthew, Alex Bateman, Cath Brooksbank, Mallory Freeberg, Melissa Harrison, Matthew Hartley, Thomas Keane, et al. 2023. “EMBL’s European Bioinformatics Institute (EMBL-EBI) in 2022.” *Nucleic Acids Research* 51: D9−D17. <https://doi.org/10.1093/nar/gkac1098>

19. Katz, Kenneth, Oleg Shutov, Richard Lapoint, Michael Kimelman, J Rodney Brister, Christopher O’Sullivan. 2022. “The Sequence Read Archive: a decade more of explosive growth.” *Nucleic Acids Research* 50: D387−D390. <https://doi.org/10.1093/nar/gkab1053>

20. Yang, Wanjuan, Jorge Soares, Patricia Greninger, Elena J. Edelman, Howard Lightfoot, Simon Forbes, Nidhi Bindal, et al. 2013. “Genomics of Drug Sensitivity in Cancer (GDSC): a resource for therapeutic biomarker discovery in cancer cells.” *Nucleic Acids Research* 41: D955−D961. <https://doi.org/10.1093/nar/gks1111>

21. Barretina, Jordi, Giordano Caponigro, Nicolas Stransky, Kavitha Venkatesan, Adam A Margolin, Sungjoon Kim, Christopher J Wilson, et al. 2012. “The Cancer Cell Line Encyclopedia enables predictive modelling of anticancer drug sensitivity.” *Nature* 483: 603−607. <https://doi.org/10.1038/nature11003>

22. Sondka, Zbyslaw, Nidhi Bindal Dhir, Denise Carvalho-Silva, Steven Jupe, Madhumita, Karen McLaren, Mike Starkey, et al. 2024. “COSMIC: a curated database of somatic variants and clinical data for cancer.” *Nucleic Acids Research* 52: D1210−D1217. <https://doi.org/10.1093/nar/gkad986>

23. Sayers, Eric W, Jeffrey Beck, Evan E Bolton, J Rodney Brister, Jessica Chan, Ryan Connor, Michael Feldgarden, et al. 2025. “Database resources of the National Center for Biotechnology Information in 2025.” *Nucleic Acids Research* 53: D20−D29. <https://doi.org/10.1093/nar/gkae979>

24. Andrews, Simon. 2023. “FastQC: a quality control tool for high throughput sequence data.” <http://www.bioinformatics.babraham.ac.uk/projects/fastqc>.

25. Dobin, Alexander, Carrie A. Davis, Felix Schlesinger, Jorg Drenkow, Chris Zaleski, Sonali Jha, Philippe Batut, Mark Chaisson, Thomas R. Gingeras. 2013. “STAR: ultrafast universal RNA-seq aligner.” *Bioinformatics* 29: 15−21. <https://doi.org/10.1093/bioinformatics/bts635>

26. Liao, Yang, Gordon K. Smyth, Wei Shi. 2014. “featureCounts: an efficient general purpose program for assigning sequence reads to genomic features.” *Bioinformatics* 30: 923−930. <https://doi.org/10.1093/bioinformatics/btt656>

27. Dyer, Sarah C., Olanrewaju Austine-Orimoloye, Andrey G. Azov, Matthieu Barba, If Barnes, Vianey Paola Barrera-Enriquez, Arne Becker, et al. 2025. “Ensembl 2025.” *Nucleic Acids Research* 53: D948−D957. <https://doi.org/10.1093/nar/gkae1071>

28. Goldfarb, Tamara, Vamsi K Kodali, Shashikant Pujar, Vyacheslav Brover, Barbara Robbertse, Catherine M Farrell, Dong-Ha Oh, et al. 2025. “NCBI RefSeq: reference sequence standards through 25 years of curation and annotation.” *Nucleic Acids Research* 53: D243−D257. <https://doi.org/10.1093/nar/gkae1038>

29. Irizarry, Rafael A., Benjamin M. Bolstad, Francois Collin, Leslie M. Cope, Bridget Hobbs, Terence P. Speed. 2003. “Summaries of Affymetrix GeneChip probe level data.” *Nucleic Acids Research* 31: e15−e15. <https://doi.org/10.1093/nar/gng015>

30. Christudas, Binildas. 2019. “MySQL.” *Practical Microservices Architectural Patterns: Event-Based Java Microservices with Spring Boot and Spring Cloud*, Apress, 877−884. <https://doi.org/10.1007/978-1-4842-4501-9_27>

31. Paul, McFedries. 2019. “Web design playground: HTML & CSS the interactive way.” *Manning*, <http://ieeexplore.ieee.org/document/10280292>

32. Badros, Greg J., Alan Borning, Kim Marriott, Peter Stuckey. 1999. “Constraint cascading style sheets for the Web.” *Proceedings of the 12th annual ACM symposium on User interface software and technology* 73−82.

33. Steven, A. Gabarro. 2007. “JavaScript - a client-side scripting language.” *Web Application Design and Implementation: Apache 2, PHP5, MySQL, JavaScript, and Linux/UNIX*, IEEE, 171−183. <https://doi.org/10.1109/9780470083963.ch14>

34. Team Ant Design. 2023. “An enterprise-class UI design language and React UI library.” <https://github.com/ant-design/ant-design>.

35. Li, Deqing, Honghui Mei, Yi Shen, Shuang Su, Wenli Zhang, Junting Wang, Ming Zu, Wei Chen. 2018. “ECharts: A declarative framework for rapid construction of web-based visualization.” *Visual Informatics* 2: 136−146. <https://doi.org/https://doi.org/10.1016/j.visinf.2018.04.011>

**Supplementary Figures**

**
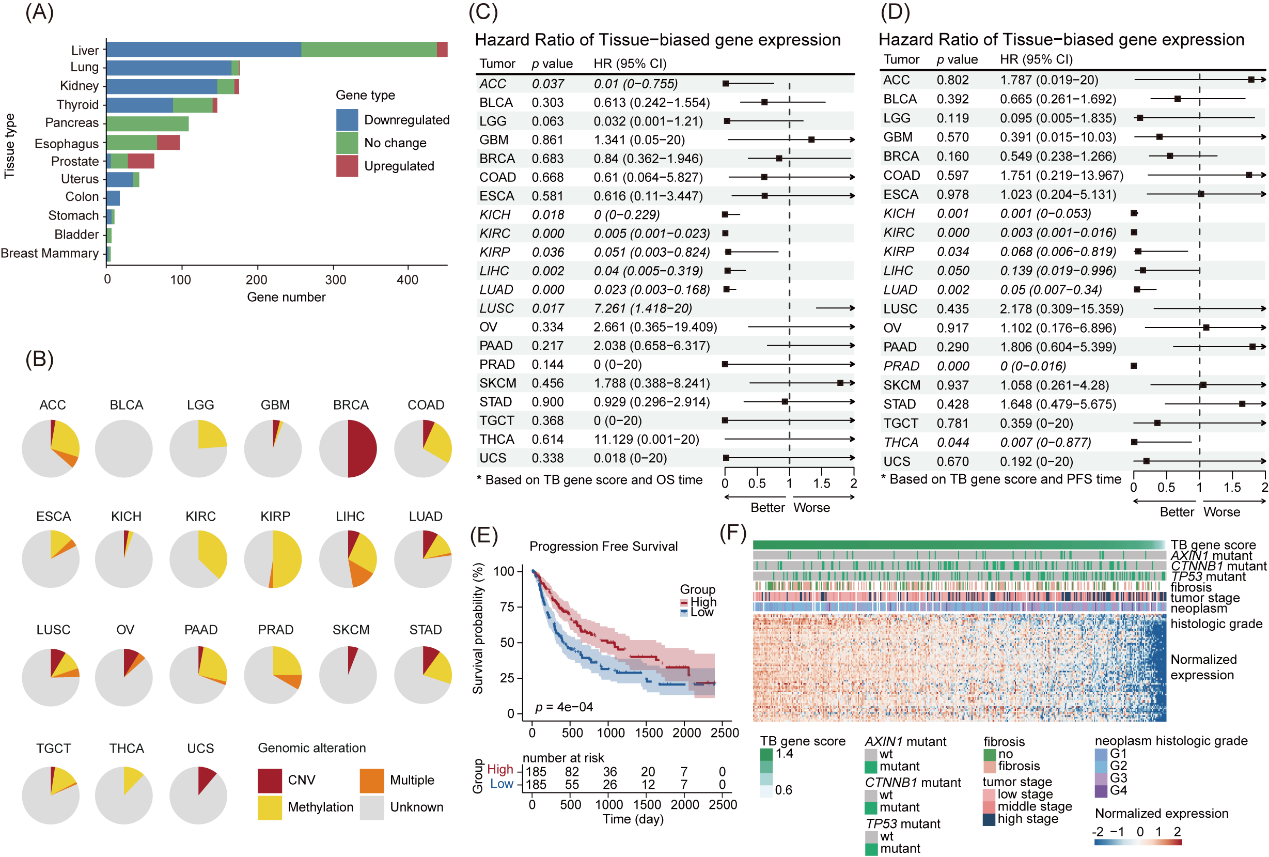
**

**Figure S1. Pan-cancer profiles of tissue-biased genes in tumors.** (A) The number of tissue-biased genes that were differentially expressed in corresponding tumor samples compared to NAT (*p* adj < 0.05, limma). (B) The proportions of tissue-biased genes whose expression were correlated with genomic alterations in each cancer type (*p* adj < 0.05, two-tailed independent *t*-test). (C−D) Forest plots showing hazard ratio (HR) for high expression of tissue-biased genes over low expression of tissue-biased genes based on overall survival (OS) and progression free survival (PFS) time. In the figures, cancer types were denoted in italics when their tissue-biased gene expression demonstrated a significant positive relationship with OS and PFS (*p* < 0.05, Cox regression). (E) Stratification of TCGA LIHC cohorts with tissue-biased gene score (median cut-off) for PFS (*p* < 0.05, log-rank test). (F) The distribution of cancer driver mutations and clinical signatures across TCGA LIHC patients ranked by the tissue-biased gene scores.

**
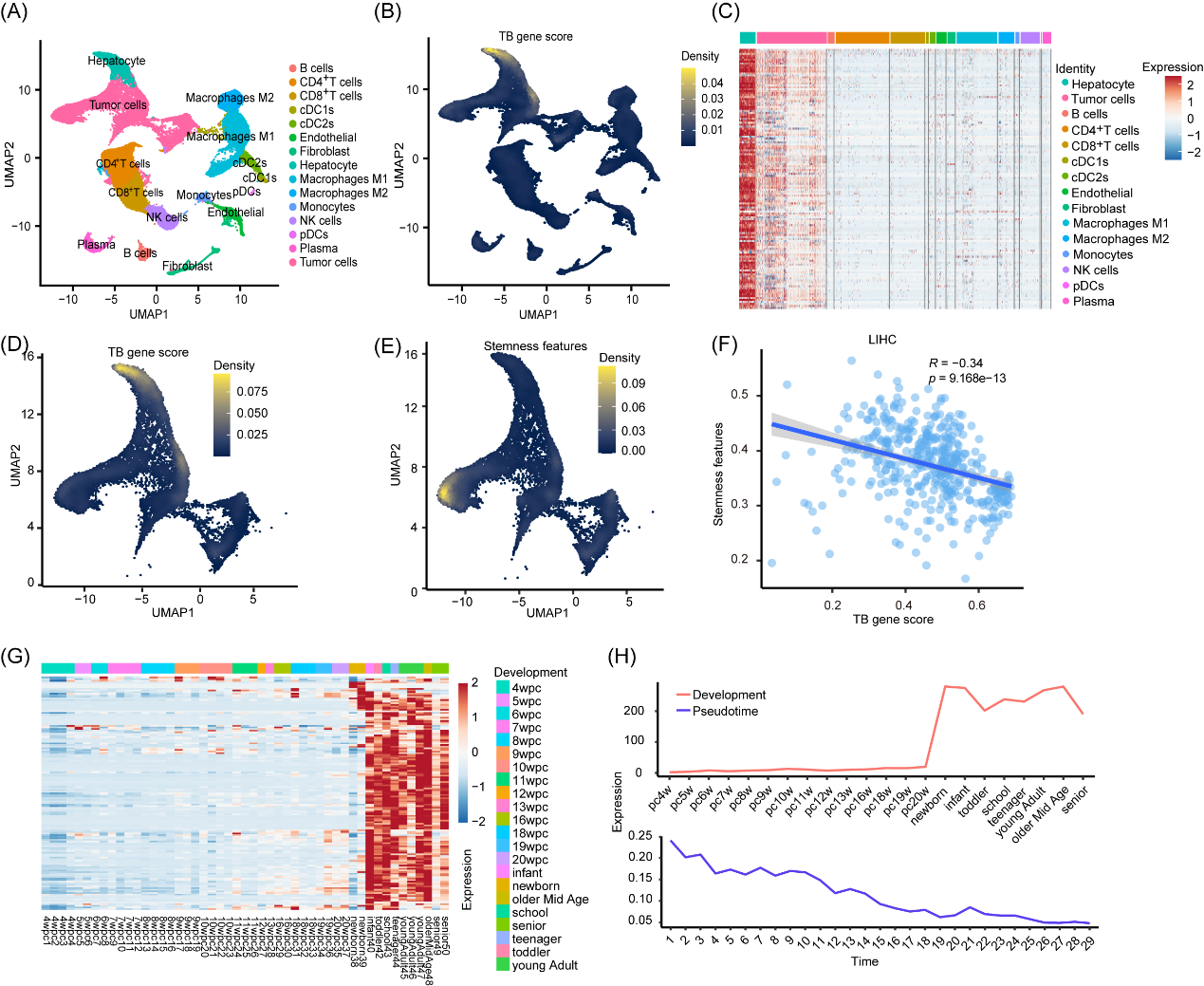
**

**Figure S2. Single-cells analysis revealed dramatic loss of tissue-biased genes during tumor evolution in LIHC.** (A) UMAP plot of all cell types annotated by unique colors in liver cancer. (B) UMAP plot of the tissue-biased gene score in all cell types. (C) Heatmap of the tissue-biased gene score after Z-Score normalization in all cell types. (D) UMAP plot of the tissue-biased gene score in different cancer cell types. (E) UMAP plot of the stemness score in different cancer cell types. (F) Correlation between tissue-biased gene scores and stemness score in liver cancer (*p* < 0.05, Pearson’s correlation coefficient). (G) Expression patterns of tissue-biased genes during embryo development and differentiation into normal liver tissue. (H) Expression changes of tissue-biased genes in embryo differentiation and tumor evolution.

**
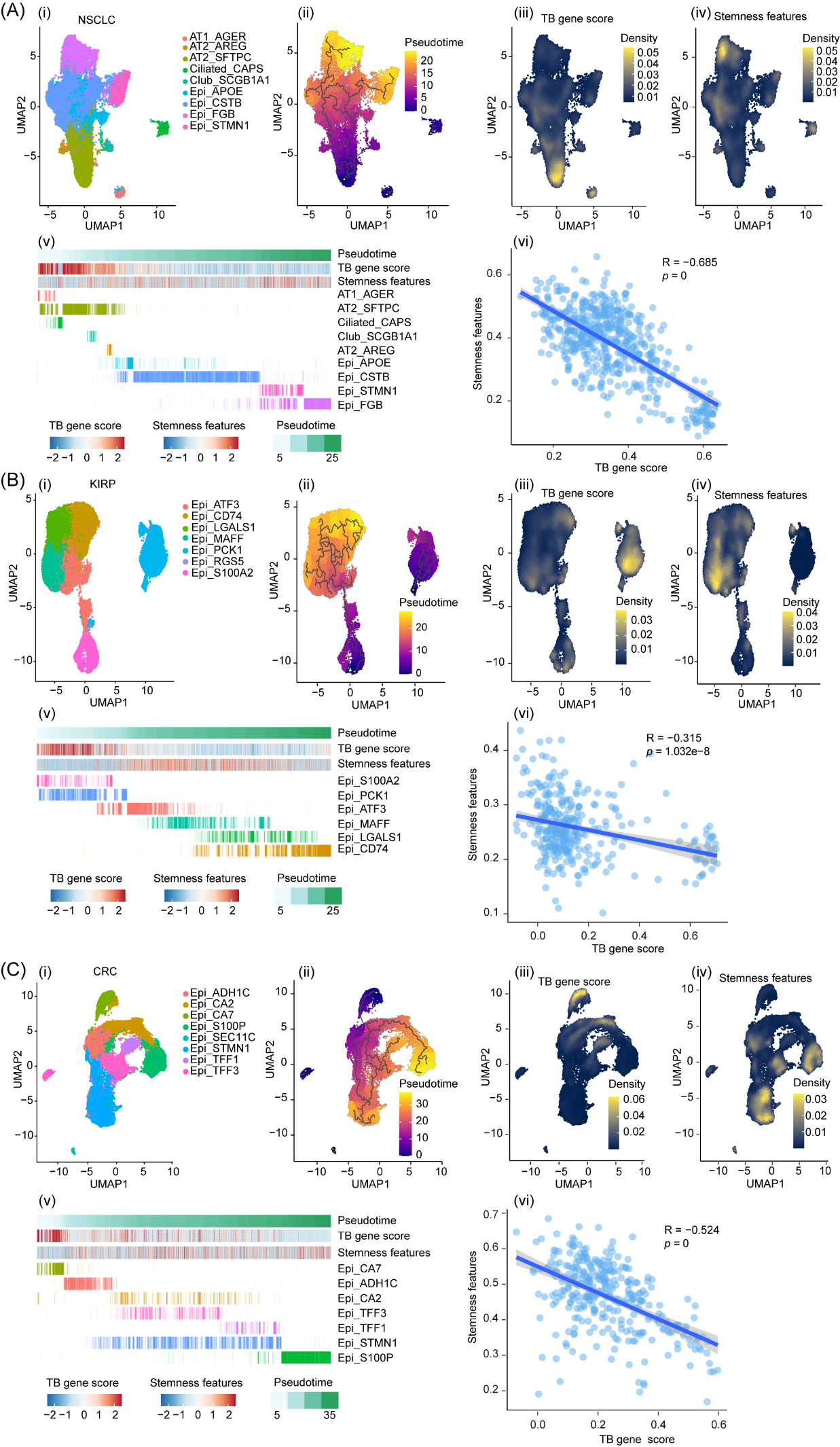
**

**Figure S3. Single-cells analysis on tissue-biased genes in** **NSCLC,** **KIRP and CRC.** Parallel analyses on (A) NSCLC, (B) KIRP, and (C) CRC showing: UMAP plot of (i) annotated cluster, (ii) pseudotime score, (iii) tissue-biased gene score, and (iv) stemness score of cancer cell types. (v) Heatmap of tissue-biased gene score and stemness score of cancer cell types at different evolutionary stages. (vi) Correlation between tissue-biased gene scores and stemness score (*p* < 0.05, Pearson’s correlation coefficient).

**
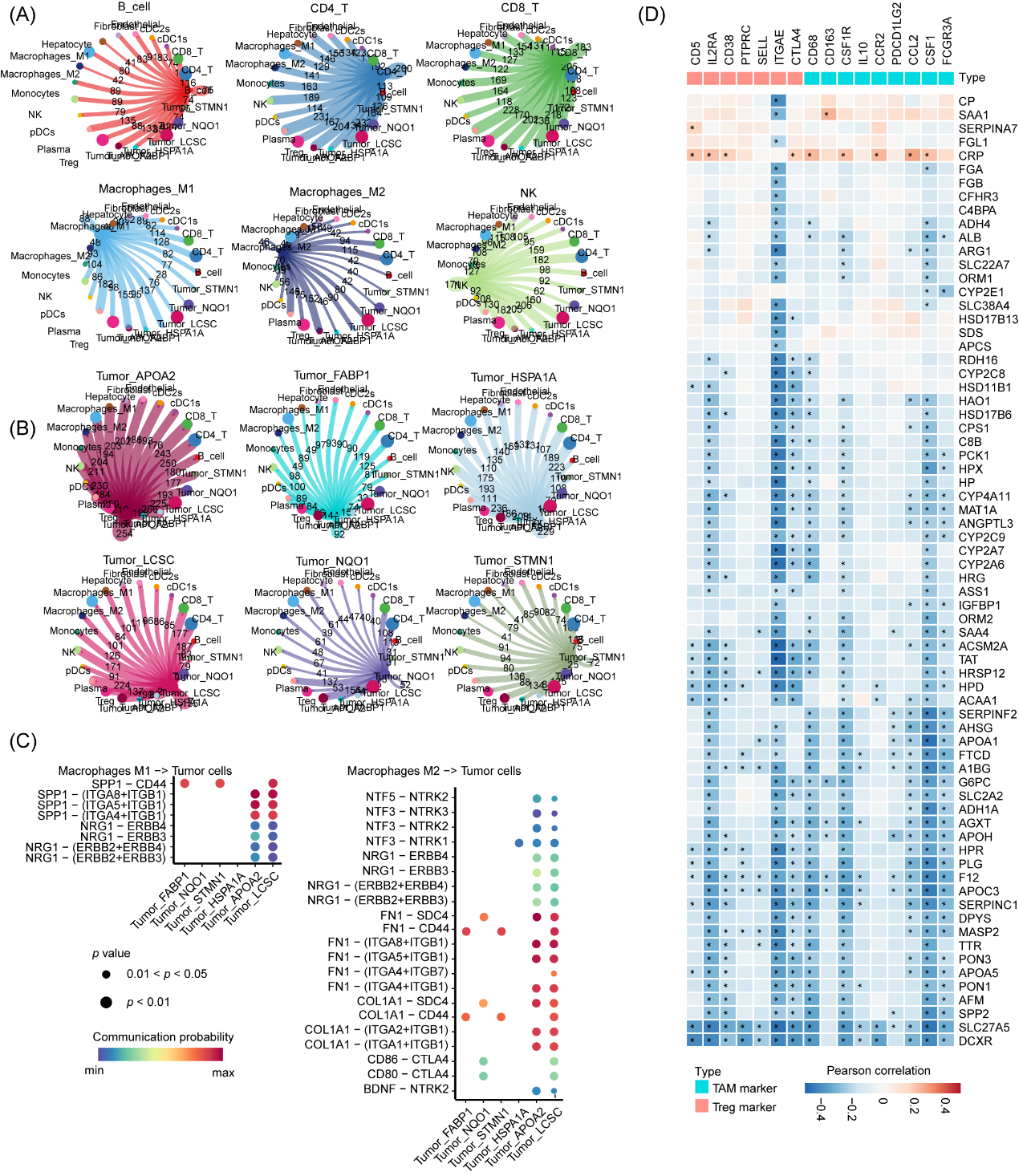
**

**Figure S4. Abnormal expression of tissue-biased genes affects immune crosstalk in tumor.** (A−B) The probability of cell communication between liver cancer cells and immune cells modeled by CellChat. (C) The significant ligand-receptor interactions between liver cancer cells and immune cells modeled by CellChat (*p* < 0.05). (D) The negative correlation between the expression of liver tissue-biased genes and Treg and TAM surface marker genes in liver cancer (*p* < 0.05, Pearson’s correlation coefficient). Statistical significance thresholds were set at **p* < 0.05, ***p* < 0.01, ****p*< 0.001, and *****p* < 0.0001.

**
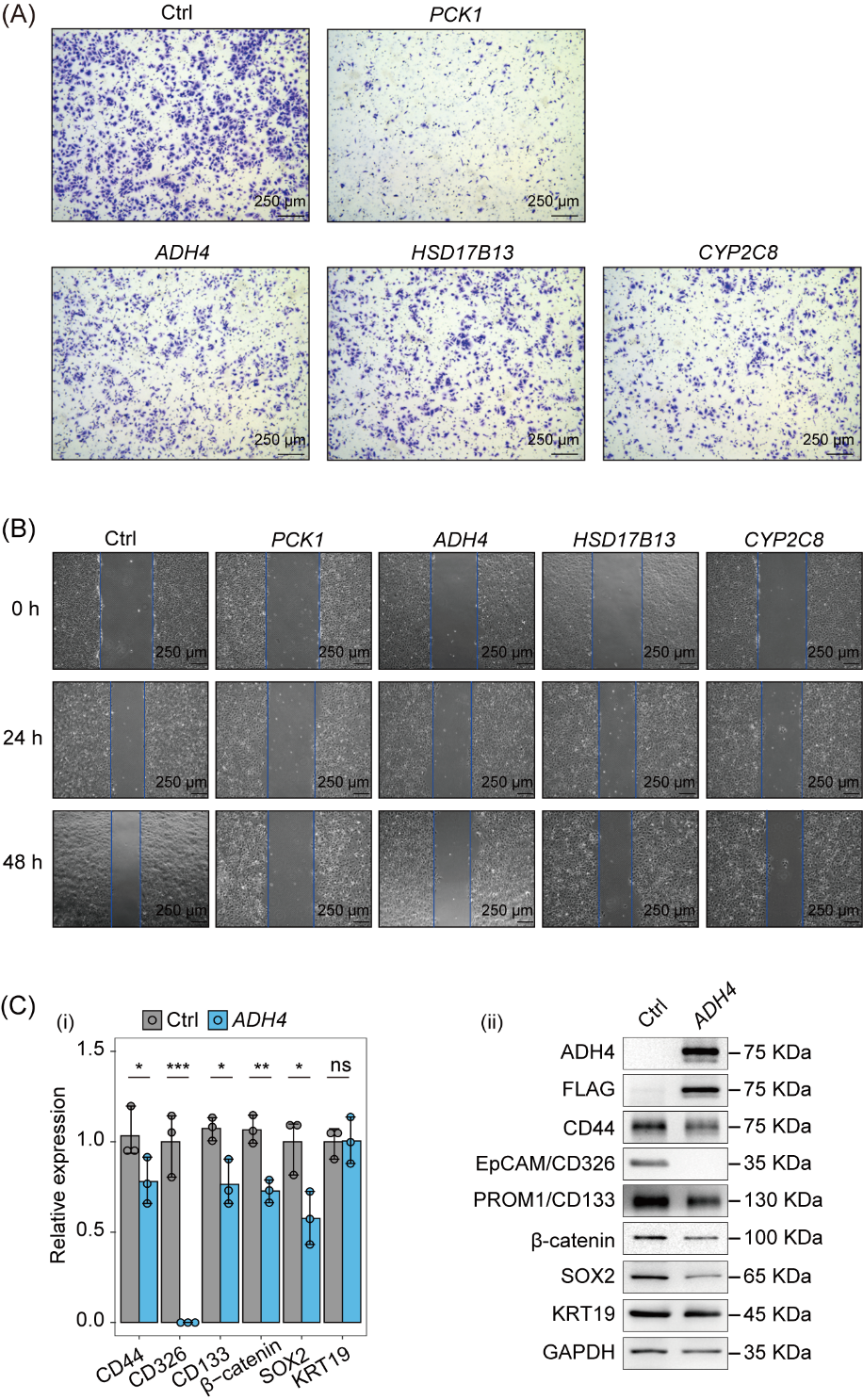
**

**Figure S5. The inactivation of tissue-biased genes in liver cancer enhances tumor stemness and affects tumor proliferation and metastatic ability.** (A) Cell migration of control and OE Huh-7 cells measured by transwell assay. (B) Cell migration of control and OE Huh-7 cells measured by scratch assay. (C) Western blotting for stemness-related proteins in control and OE-*ADH4* Huh-7 cells included (i) the relative band intensity compared to GAPDH control in each condition and comparative analysis of expression levels from triplicate experiments (*p* < 0.05, two-tailed Mann-Whitney *U* test) and (ii) representative blots. Statistical significance thresholds were set at ns not significant, **p* < 0.05, ***p* < 0.01, ****p*< 0.001, and *****p* < 0.0001.

**
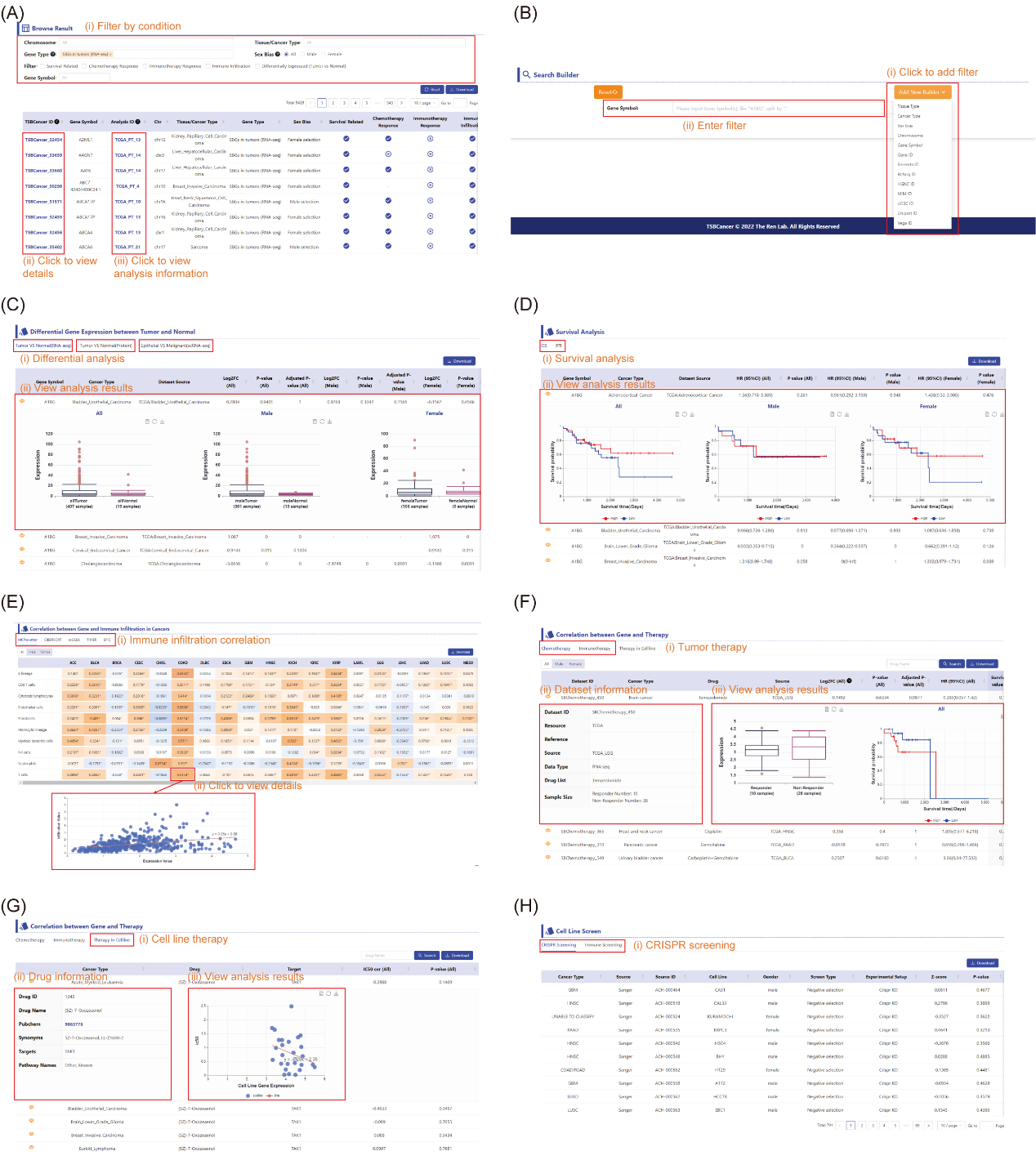
**

**Figure S6. The function and usage of TBCancer.** (A−B) The browsing and search function. (C) Module for differential analysis of biased genes expression between tumors and corresponding normal tissues. (D) Survival analysis module of biased genes in cancer. (E) Correlation analysis module of biased genes and tumor immune infiltration. (F) Analysis module of biased genes in tumor therapy. (G) Correlation analysis module between biased genes and tumor drug IC_50_. (H) CRISPR-Screen and immune CRISPR-Screen modules of biased genes.
